# Patient-derived gastric cancer organoids model heterogeneity and stroma-mediated chemoresistance in poorly cohesive carcinoma

**DOI:** 10.3389/fmolb.2025.1631168

**Published:** 2025-06-30

**Authors:** Yi Yin, Shuhong Zeng, Hui Zhang, Shuangshuang Wang, Fei Ke, Qiang Rui, Jinyong Zhou, Yuwen Zhuang, Weixing Shen, Jun Qian, Shenlin Liu

**Affiliations:** ^1^ Department of Oncology, Affiliated Hospital of Nanjing University of Chinese Medicine, Jiangsu Province Hospital of Chinese Medicine, Nanjing, Jiangsu, China; ^2^ No. 1 Clinical Medical College, Nanjing University of Chinese Medicine, Nanjing, Jiangsu, China; ^3^ Department of Chinese Medicine, Affiliated Changshu Hospital of Nantong University, Changshu No.2 People’s Hospital, Changshu, Jiangsu, China; ^4^ Department of Pathology, Affiliated Hospital of Nanjing University of Chinese Medicine, Jiangsu Province Hospital of Chinese Medicine, Nanjing, Jiangsu, China; ^5^ Department of General Surgery, Affiliated Hospital of Nanjing University of Chinese Medicine, Jiangsu Province Hospital of Chinese Medicine, Nanjing, Jiangsu, China; ^6^ Central Laboratory, Affiliated Hospital of Nanjing University of Chinese Medicine, Jiangsu Province Hospital of Chinese Medicine, Nanjing, Jiangsu, China; ^7^ Jiangsu Collaborative Innovation Center of Traditional Chinese Medicine Prevention and Treatment of Tumor, Nanjing, Jiangsu, China

**Keywords:** gastric cancer (GC), patient-derived organoids, poorly cohesive carcinoma (PCC), drug sensitivity, tumor microenvironment

## Abstract

**Objective:**

Gastric cancer (GC) remains a leading cause of cancer-related mortality, with poorly cohesive carcinoma (PCC) exhibiting rising incidence and poor therapeutic responses. In this study, we constructed a panel of patient-derived organoids of GC and used them to understand histological accuracy and sensitivity profiles as well as the influence of microenvironment on these special GC subtypes.

**Methods:**

A total of 23 patient-derived GC organoid models including 12 PCC and 11 non-poorly cohesive carcinoma (NPCC) were established. Histopathological and genetic fidelity to primary tumors was validated using immunohistochemistry (IHC), immunofluorescence (IF), and H&E staining. Relative growth kinetics between PCC and NPCC organoids were measured and compared. Sizes of subcutaneously xenografted tumors harbored by mice representing different types of GC organoids were analyzed. We further examined drug sensitivities between docetaxel, 5-fuorouracil (5-FU), oxaliplatin, and irinotecan against our collected samples of patient-derived organoids. The impact of cancer-associated fibroblasts (CAFs) on organoid growth and chemoresistance was analyzed via co-culture experiments.

**Results:**

Organoids retained genetic and histopathological features of primary tumors. PCC-derived organoids displayed rapid growth characteristics *in vitro* and produced more rapidly growing tumors *in vivo* than NPCC. PCC organoids showed heightened sensitivity to docetaxel with lower IC_50_, but no significant differences were observed for 5-FU, oxaliplatin, or irinotecan. CAF co-culture enhanced organoid proliferation and conferred resistance to all tested chemotherapeutic agents.

**Conclusion:**

Patient-derived GC organoids especially PCC subtypes reliably recapitulate the complexity of solid-tumors heterogeneity, predict drug responses, and elucidate stromal contributions to therapy resistance. This model could be used to develop tailoring treatments and improve personalization of therapy for clinical management of PCC.

## 1 Introduction

Gastric cancer (GC) ranks fifth and third in the world in terms of cancer incidence and death, respectively (Smyth et al., 2020). Although the overall trend of worldwide GC incidence has decreased recently, the relative incidence of poorly cohesive carcinoma (PCC) continued to rise steadily (Drubay et al., 2022). The clinical relevance of PCC lies in that early extensive submucosal invasion, lympho-vascular permeation, peritoneal dissemination made patients could not accept curative surgery after diagnosis. In addition, it often occurred at younger ages than intestinal type GC and frequently caused disproportionate morbidity in young people. More importantly, due to its inherent resistance to routine cytotoxic chemotherapy regimens, most PCCs showed extremely poor survival prognosis with a 5-year survival rate much lower than that of intestinal type counterparts matched by stage (Nakamura et al., 2022). Therefore, this subtype exhibits limited response rates to conventional therapies, with significant inter-individual variability. Enhancing the effectiveness of clinical interventions necessitates the development of precise predictive tools for chemotherapy outcomes (European Chapter of International Gastric Cancer Association et al., 2019).

Tumor organoids derived from patient cancer cells via *in vitro* three-dimensional (3D) culture served as an exact miniature replica of the tumor (Xu et al., 2022). These organoids faithfully reflected the genetic traits, genetic heterogeneity, tissue structures and functions of primary tumors and faithfully recapitulated genetic profiles, histological characteristics and other pathomorphologic features based on the same donor (Veninga and Voest, 2021). This fidelity suggests their promise for personalized cancer therapy (Lv et al., 2024). Several teams got the organoids from different histology and clinical stages of GC for drug testing preclinical model (Huo et al., 2023). Nonetheless, there are few reports about generating organoids from PCC.

We successfully built 23 cases of GC organoid models based on patient tissues, divided into PCC group and NPCC group according to the absence or presence of PCC components, respectively. We performed phenotypic and molecular characterization including pathological appearance and expression of clinicopathological markers on GC organoids and related original tumor tissue, separately. Afterward, we used organoids derived from patients for xenografting, then we did drug sensitivity test on them using four kinds of standard chemotherapy drugs. Finally, we prepared the co-culture system between GC organoids and associated tumor-associated fibroblasts as a tumor microenvironment and drug sensitivity tested too.

## 2 Materials and methods

All the antibodies and reagents used in this study were listed in Supplementary Table S1, including dilution ratio and drug concentration.

### 2.1 Samples and patients

We collected fresh GC tissues from patients of Jiangsu Province Hospital of Chinese Medicine that had surgery to remove tumors. Organoids were kept in the recovery solution (Bio Genous Technology, E238006). Patient-specific baseline attributes such as histological classification, cancer stage, and therapeutic interventions were documented. Histopathological analysis revealed adenocarcinoma, comprising 13 cases of PCC and 14 cases of non-poorly cohesive carcinoma (NPCC).

### 2.2 Establishment of patient-derived gastric cancer (GC) organoids

After sample collection, the tissue was washed 10 times in PBS solution containing penicillin-streptomycin-gentamicin (1%, 100×, Solarbio P1010), dissociated using sterile surgical scissors to cut into tissue particles 2–3 mm^3^ and digested with Tumor Tissue Dissociation Kit (Bio Genous Technology, K601003-A100/K601008-B100) at 37°C for 30 min while gently shaking (Ouyang et al., 2022).

Filtered in series on 70 μm nylon gauze (Falcon, 352,350), spun down (250 × g for 3min at 4°C). If there are red pellet after spinning, then lysis needs to be done using Red Blood Cell Lysis Buffer (Bio Genous Technology, E238010) as previously described (Tong et al., 2023).

Cell pellets were subsequently resuspended in Growth Factor-Reduced Matrigel (Corning, 356,231) at a density of 1 × 10^4^ cells/50 μL and plated in 48-well culture plates (Corning, 3,548). Following matrix polymerization (10 min at 37°C, 5% CO_2_), GC organoid culture medium (Bio Genous Technology, K2179-GC-A500) was carefully overlaid. Culture maintenance included medium replacement every 72–96 h and routine passaging every 7–14 days using Organoid Dissociation Reagent (Bio Genous Technology, E238001) (Yu et al., 2023).

### 2.3 Isolation and culture of cancer-associated fibroblasts (CAFs)

Harvested tumor tissues were placed in a 6 cm culture dish, mechanically chopped into pieces of 1 mm, and gently shaken and digested at 37°C for 1 h. We made the digesting solution with phosphate-buffered saline containing calcium and magnesium ions, 1 mg/mL collagenase Ⅳ, and 1 mg/mL DNase Ⅰ. Tumor tissue homogenate was centrifuged for 7–10 min at 4°C and 350× g before being rinsed three times with DMEM medium containing 10% FBS. After digestion, tumor homogenates were passed through a 70 μm strainer and rinsed with new PBS containing 2% FBS. The cell suspension was centrifuged for 7–10 min at 4°C and 350× g, followed by erythrocyte lysis. In summary, pellets were resuspended with 1 mL of lysis buffer and agitated for 1 min. Then, PBS containing serum was added and the suspension was centrifuged for 7–10 min (4°C, 350× g). We resuspended the cell pellets in 5 mL of fresh PBS containing FBS and counted them using 0.4% trypan blue solution (w/v).

### 2.4 Immunohistochemical (IHC) staining and hematoxylin-eosin (H&E) staining

IHC staining was performed on 4 μm FFPE sections following antigen retrieval in citrate buffer (10 mM, pH 6.0, 95°C, 20 min). After peroxidase blocking and serum incubation, sections were treated with primary antibodies (dilutions in Supplementary Table S1) at 4°C overnight, followed by HRP-conjugated secondary antibodies (1:500) and DAB visualization. Two blinded pathologists independently scored staining intensity (0–3) and positive area (0–4), with composite H-scores (0–12) calculated as intensity × percentage. Digital imaging was conducted using a Nikon Eclipse Ni-E microscope (200×, DS-Fi3 camera) (Steele et al., 2019).

Tissues fixed in 4% paraformaldehyde underwent graded ethanol dehydration, paraffin embedding, and sectioning (4 μm). Sections were dewaxed in xylene, rehydrated through ethanol gradients, acid-conditioned (5% acetic acid, 1 min), and sequentially stained with Mayer’s hematoxylin (5 min, 25°C) and eosin (1 min). After ethanol-xylene dehydration, slides were mounted with neutral resin for histomorphological analysis using an Olympus IX81 microscope (DP74 camera).

### 2.5 Immunofluorescence staining

Following experimental interventions, organoids in 48-well microplates were collected and rinsed thrice with ice-cold PBS, prior to fixation with 4% paraformaldehyde and then permeabilized with 0.3% Triton X-100 at room temperature for 30 min. Following the application of Immunol staining blocking buffer, the cells were incubated overnight at 4°C with primary anti-CDX-2 and anti-CEA antibodies, then incubated with coupled CoraLite®488-conjugated or CoraLite®594-conjugated secondary antibodies (Koh et al., 2021). The nuclei were stained with DAPI. Organoid were imaged in *z*-axis steps of 2 mm with a laser confocal microscope (FV4000, Olympus, Tokyo, Japan).

### 2.6 Establishment of co-culture units

A direct contact co-culture model was established to investigate cellular interactions between GC organoids and cancer-associated fibroblasts (CAFs), labeling by different fluorescence (GC cells: mCherry, MSCs: GFP). Human CAFs were isolated from GC tissues following established protocols (Zhao et al., 2022). CAFs used for experimental studies at passages 4–6. CAFs and organoids at a ratio of 1:2 embedded in Matrigel (Zhao et al., 2024). After 5 days of co-culture, the co-culture state was photographed in *z*-axis steps of 2 mm with a laser confocal microscope (FV4000, Olympus, Tokyo, Japan).

### 2.7 Organoid-based xenotransplantation

Nude mice (BALB/c-nu/nu) aged 4–6 weeks were procured from Beijing Vital River Laboratory Animal Technology Co., Ltd. All experimental animals were free of specific pathogens. Patient 1- and patient 8-derived organoids were retrieved using organoid recovery solution (Bio Genous Technology, E238006). A specific quantity of organoids was resuspended in 50% Matrigel based on the number of mice available. Subsequently, 100 μL of the mixture was subcutaneously injected into the right forelimb of nude mice (Qu et al., 2021). Regular weighing of the mice was conducted, and measurements of tumor length and width were taken to determine tumor volume (tumor volume = length × width × width/2). The experiment was terminated if the tumor diameter exceeded 20 mm or if significant weight loss was observed, at which point the mice were euthanized (Yoshida, 2020).

### 2.8 Organoid *in vitro* drug screens

Three days after seeding, the culture medium was replaced with media containing varying concentrations of 5-fluorouracil (5-FU), oxaliplatin, irinotecan, and docetaxel. Following a 72-h incubation period, the half-maximal inhibitory concentration (IC_50_) was determined to assess drug sensitivity (Li et al., 2022).

### 2.9 Statistical analysis

All experimental procedures were independently replicated three times in triplicate. Quantitative data were presented as mean ± standard error of the mean (SEM) and demonstrated normal distribution. Intergroup comparisons were conducted using T-tests or one-way ANOVA, with the choice of *post hoc* testing determined by variance homogeneity. Post hoc analyses were performed using either Tukey’s multiple comparison test or Dunnett’s T3 test. Statistical analyses were carried out using GraphPad Prism® software (GraphPad Inc., USA). Statistical significance was defined as *P* < 0.05 for all tests. The statistical staff performed analyses in a blinded manner.

## 3 Results

### 3.1 Human GC organoids are established from cancer tissues

**FIGURE 1:**
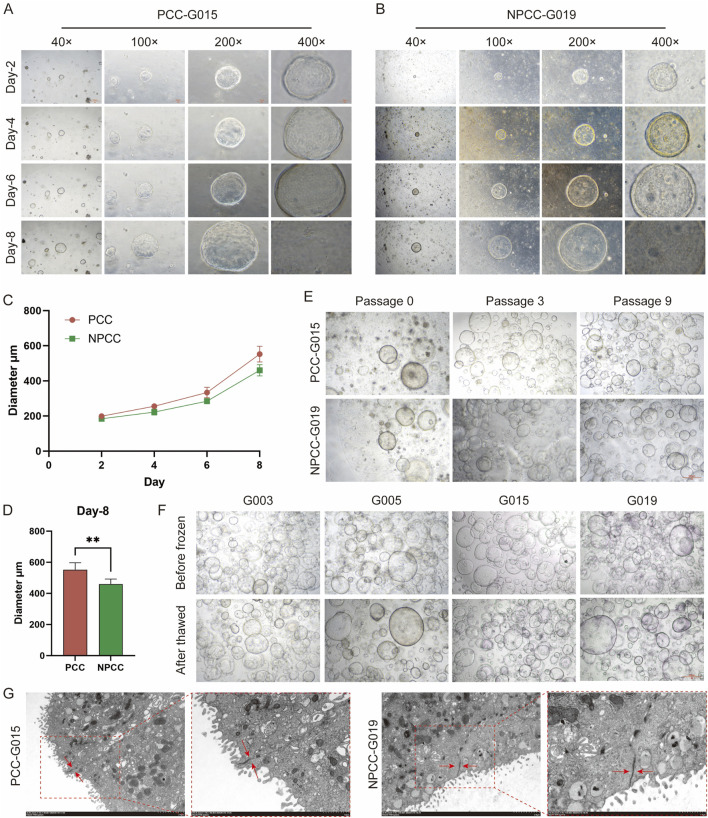
Bright field performance and growth characteristics of GC organoids. **(A,B)** The growth conditions and processes of PCC and NPCC organoids at different magnifications, scale bar,500, 200, 100, and 50 μm, respectfully. **(C)** Diameter growth curves of PCC and NPCC organoids. **(D)** Diameter growth curves of PCC and NPCC organoids. **(E)** Diameters of PCC and NPCC organoids on the eighth day after passage. Long-term culture characteristics of PCC and NPCC organoids, scale bar, 500 μm. **(F)** Bright-field images of PCC and NPCC organoids before cryopreservation and after resuscitation, scale bar, 500 μm. **(G)** Transmission electron microscope images of PCC and NPCC organoids, scale bar, 2.0 μm and 1.0 μm, respectfully. Data are presented as mean ± standard deviation. *P < 0.05, **P < 0.01.

With the aid of 3D organoid culture technique, we successfully built 23 GC organoid lines out of 27 tissue specimens (Construction efficiency: 85.19%) (Table 1). The Inclusion/exclusion criteria for participants and sample collection rules are listed in Supplementary Material S1. The cohort comprised 11 NPCC and 12 PCC cases, with success rates of 78.57% (11/14) and 92.31% (12/13), respectively. Clinical and pathological characteristics of the samples are detailed in Supplementary Table S2. Notably, organoid culture success rates showed no correlation with clinical characteristics. After 3D culturing within several days, GC organoids developed circular cavity-like structures. To evaluate organoid growth dynamics, we conducted longitudinal morphological analyses (Figures 1A,B). After 7 days of culture, individual organoids attained diameters of up to 500 μm, with PCC-derived organoids exhibiting significantly faster growth rates and larger sizes compared to NPCC organoids (Figures 1C,D). Following successful establishment, both PCC and NPCC organoids demonstrated long-term culture viability while maintaining stable morphological characteristics (Figure 1E). Furthermore, cryopreservation and consequent revival of organoids did not affect significant difference among morphological attributes nor changed related to overall phenotypic features or appearances. Transmission electron microscopy revealed that the organoids formed spherical hollow structures consisting of tightly adherent cells (Liu et al., 2024b), where intercellular contacts were dominated by tight junction morphology (Figure 1G).

**TABLE 1:** Baseline characteristics of the patients.

Characteristics	Total	Organoids culture	
	27	Success (23)	Fail (4)
Sex			
Male	21	17	4
Female	6	6	0
Age			
>60	21	18	3
<60	6	5	1
Tumor Size			
<5	18	14	4
>5	9	9	0
Tumor Histological Morphology			
NPCC	14	11	3
PCC	13	12	1
Tumor differentiation			
G1	2	1	1
G2	11	9	2
G3	14	13	1
Lauren type			
Intestinal	10	8	2
Diffuse	10	10	0
Mixed type	7	5	2
LVI			
Yes	20	17	3
No	7	6	1
PNI			
Yes	20	17	3
No	7	6	1
TNM stage			
I	4	4	0
II-III	23	19	4

### 3.2 GC organoids maintain the histology of original cancer tissues

**FIGURE 2:**
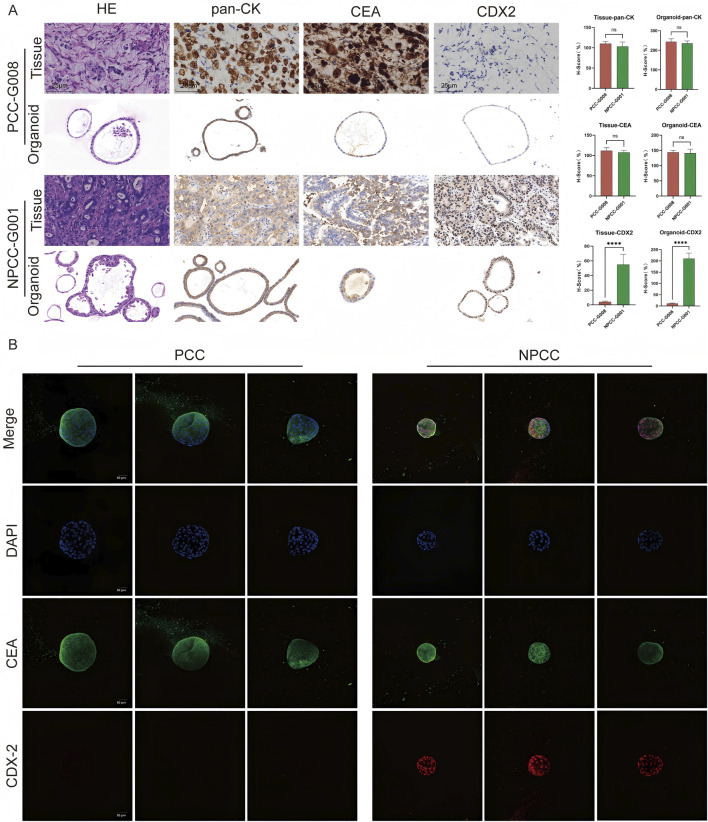
GC organoids can reproduce the histological characteristics of the primary tissue. **(A)** H&E and IHC staining (pan-CK, CEA, CDX-2) revealed that the PCC organoids successfully cultured exhibited histological features consistent with the primary tumor but distinct from NPCC, scale bar, 500 μm. The H-socre statistics of the corresponding indicators were also displayed. Data are presented as mean ± standard deviation. ****P < 0.0001. **(B)** Immunofluorescence analysis demonstrated similar CEA expression (green) in both PCC and NPCC organoids, whereas CDX-2 expression (red) differed between them.

We conducted hematoxylin and eosin (H&E) staining and immunohistochemistry (IHC) analyses on the matched organoids and tissues (Figure 2A). Immunofluorescence (IF) was utilized to examine the expression of the typical GC markers CEA and CDX-2 (Figure 2B). The results show that the level of pan-CK and CEA was high expression in GC tissue and organoids, but opposite, CDX-2 displayed in NPCC tissue and organoids with low level in PCC tissue and organoids. The HE and IHC staining of rest PCC organoids were in Supplementary Figure S1, and NPCC organoids in Supplementary Figure S2.

### 3.3 Tumorigenicity analysis of GC organoids in nude mice

**FIGURE 3:**
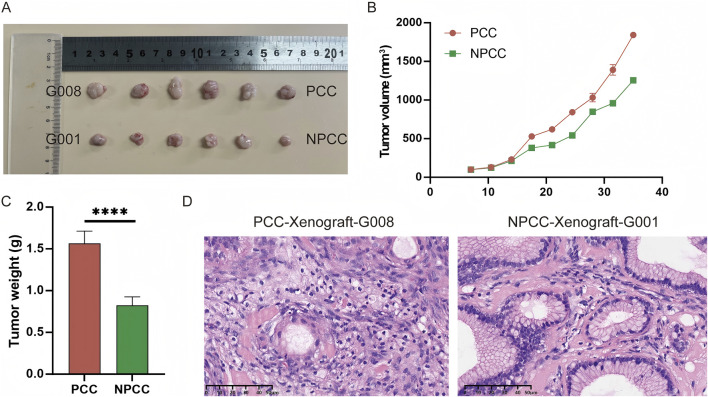
Tumorigenesis of the GC organoids *in vivo*. **(A)** Appearance of tumors formed subcutaneously by PCC and NPCC organoids in immunodeficient mice. **(B)** Comparison of growth curves of ectopically implanted tumors of PCC and NPCC organoids. **(C)** Comparison of weights of ectopically implanted tumors of PCC and NPCC organoids. Data are presented as mean ± standard deviation. ****P < 0.0001. **(D)** HE staining results of typical PCC and NPCC organoid-based xenotransplantation.

To assess the tumorigenicity of GC organoids, we transplanted the typical PCC and NPCC organoids into nude mice and monitored their growth. After about 40 days, the organoids formed subcutaneous tumors in the mice. The subcutaneous xenografts of PCC organoids were larger than those of NPCC (Figures 3A–C). HE staining results of organoid-based xenotransplantation were also presented (Figure 3D).

### 3.4 GC organoids for patient-specific drug trials *in vitro*

**FIGURE 4:**
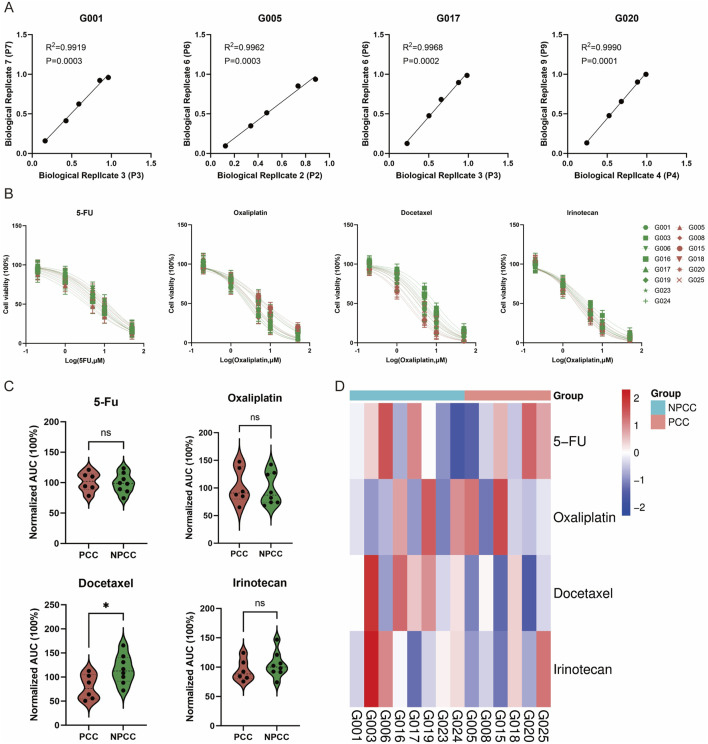
Drug screening of GC organoids to chemotherapeutics. **(A)** Scatter plots illustrate the correlation between two independent drug screening dose-response curves for the same organoid, quantified by Pearson *R* ^2^ values. **(B)** Dose-response curves depict the chemosensitivity of 6 PCC organoids compared to 8 NPCC organoids against four conventional chemotherapy agents (5-FU, oxaliplatin, docetaxel, irinotecan) (*n* = 3). **(C)** Violin plots represent the AUC of response data. **(D)** A heat map displays the AUC for four chemotherapeutic drugs across 6 PCC and 8 NPCC organoids.

GC organoids of distinct histological subtypes were assessed for *in vitro* sensitivity to four chemotherapeutic agents: 5-FU, oxaliplatin, docetaxel, and irinotecan. Two organoid cohorts, including 8 NPCC and 6 PCC cases, were treated with fixed drug concentrations (0.2, 1, 5, 10, 50 μmol/L). Dose-response curves from biological replicates of the same organoid line showed high correlation (Pearson’s *R* ^2^ > 0.99), confirming culture system stability and consistency across passages (Figure 4A). Organoids from different GC patients demonstrated varied responses to the drugs. No significant differences in half-maximal inhibitory concentration (IC_50_) values for 5-FU, oxaliplatin, and irinotecan were found between NPCC and PCC organoids. However, PCC organoids had lower IC_50_ values for docetaxel, indicating greater sensitivity to this treatment (Figures 4B,C). The response of the two groups of organoids to different drugs was presented by heatmap (Figure 4D). In a word, PCC organoids showed heightened sensitivity to docetaxel *in vitro* with lower IC_50_.

### 3.5 The organoid model as a platform to investigate interaction between cells of the tumor microenvironment and tumor cells

**FIGURE 5:**
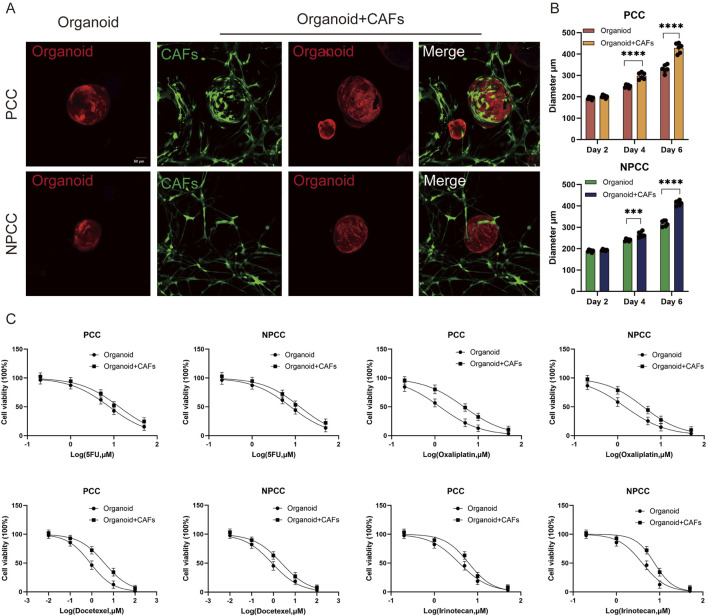
The organoid model as a platform to investigate interaction between cells of the tumor microenvironment and tumor cells. **(A)** Representative images depict co-culture of CAFs (green) with gastric cancer organoids. **(B)** Organoid diameters in both PCC and NPCC co-culture groups exceeded those in single-culture systems. **(C)** Dose-response curves compare chemotherapeutic drug sensitivity between organoid single-culture and organoid-CAFs co-culture systems (*n* = 3).

To investigate the potential for organoid-fibroblast co-culture, we established co-cultures of GC organoids (PCC-G008 and NPCC-G001) with CAFs. After 5 days of co-culture, CAFs formed an extensive network surrounding the organoids (Figure 5A). Notably, co-culture with CAFs significantly enhanced organoid growth, as evidenced by increased organoid size in both PCC and NPCC lines (Figure 5B).We further examined the influence of CAFs on organoid response to four chemotherapeutic agents (5-FU, oxaliplatin, docetaxel, and irinotecan). Organoids co-cultured with CAFs exhibited significantly greater chemoresistance compared to organoids cultured alone (Figure 5C). These findings demonstrate that CAFs modulate organoid sensitivity to chemotherapy.

## 4 Discussion

Despite its clinical aggressiveness, the underlying molecular basis responsible for the extremely invasive and therapeutically resistant behavior of PCC remains elusive, which hampers the development of clinically effective targeted therapies. We need an experimentally more clinical-like PCC model.

Our study succeeded in deriving PCC-derived GC organoids. Given the lack of preclinical models for this invasively behaved subtype, establishing this type of PCC organoid is essential. Our findings demonstrated that PCC organoids mimicked histological and molecular features of the parent tumors and validated the usefulness of these cultures for drug testing and microenvironmental studies. The rapid growth of the PCC organoids within and xenotransplantation was similar to what had been reported in the clinic about the aggressive subtype of PCC. This finding might suggest some intrinsic proliferative advantage or altered signals. Notably, the heightened sensitivity of PCC organoids to docetaxel highlights potential subtype-specific vulnerabilities, possibly linked to microtubule dynamics or differential expression of drug targets (Chen et al., 2014).

The co-culture system with CAFs has emerged as a powerful tool in oncology research, which reveals a highly significant stromal role in modulating tumor treatment resistance, consistent with previous studies implicating enhanced tumor growth and drug tolerance in the presence of CAFs. Besides, the need to incorporate stromal components in preclinical models is highlighted to better mimic *in vivo* conditions (Chen et al., 2021). For instance, a research established direct 3D co-cultures of primary pancreatic ductal adenocarcinoma (PDAC) organoids and patient-matched CAFs. They observed that upon co-culture with CAFs, there was increased proliferation and reduced chemotherapy in tumor cells (Schuth et al., 2022). Similar results were also found in breast cancer (Fan et al., 2024). Besides, A study found that CAF-derived conditioned media could induce breast cancer’s cell growth and radio-resistance through secreted interleukin six and activated the signal transducer and activator of transcription 3 (STAT3) signaling pathway (Guo et al., 2023). These results emphasize the importance of targeting tumor-stroma interactions to overcome therapeutic resistance (Zhang et al., 2023). Our co-culture system can help research on communication between tumor cells and stroma.

Although it's just preliminary data based on limited numbers, there’re limitations of our work that multi-omics profiling should be conducted further to figure out how PCC behaves uniquely (Liu et al., 2024a). herefore, genomic and transcriptomic analysis will be needed to reveal drivers of PCC aggressiveness and docetaxel response, then validation in PDX models or clinical trials would support their translation into reality.

## 5 Conclusion

In conclusion, our work establishes PCC organoids as a robust tool for personalized therapy prediction and microenvironment studies (Polak et al., 2024). By directly bridging high-fidelity histopathology with functional drug response profiling, the PCC organoid system provides a tangible pathway to inform and refine individualized treatment decisions for GC patients. Critically, this approach offers a powerful tool to guide therapeutic selection, particularly for patients with aggressive poorly cohesive subtypes, thereby advancing the paradigm of precision oncology in GC management.

## Data Availability

The raw data supporting the conclusions of this article will be made available by the authors, without undue reservation.
