# Supplementary material for: Patient-derived gastric cancer organoids model heterogeneity and stroma-mediated chemoresistance in poorly cohesive carcinoma
Source: Front Mol Biosci. 2025 Jun 30;12:1631168. doi: 10.3389/fmolb.2025.1631168 (PMC12256257; doi:10.3389/fmolb.2025.1631168)
Supplement: Supplementary file 2 [file Supplementaryfile1.docx]

**Supplement S1**

**Inclusion/exclusion criteria for participants and Sample Collection**

This study included 27 patients who received surgical treatment for gastric cancer in Jiangsu Province Hospital of Traditional Chinese Medicine between October 9, 2024 and March 24, 2025 without preoperative radiotherapy and chemotherapy.Patient-derived samples were obtained and stored in accordance with the approved procedures. The International Union Against Cancer's recommended categories for tumor grade and stage were used. Prior to analysis, all patient records and data were de-identified and anonymized. Samples were immediately stored in primary tissue storage solution at 4 ℃.

Inclusion requirements: (1) Clinical examination, gastroscopy, magnetic resonance imaging (MRI), PET-CT/CT, and hematological were used to diagnose all patients with GC for the first time. All patients' resected samples were verified by pathological analysis. (2) Patients with GC who were admitted to the Jiangsu Province Hospital of Traditional Chinese Medicine between October 9, 2024 and March 24, 2025. Additionally, pathology samples from every patient were saved for two separate specialists to validate their re-diagnosis independently. (3) Complete clinical data were available for every patient.

The following patients were disqualified: (1) Patients with a pathological diagnosis other than GC, such as a gastric stromal tumor; (2) Patients who passed away during treatment; (3) Patients with incomplete data or who lost to follow-up; (4) Patients with an infection, an immune system disorder, a blood system condition; (5) Patients who had local radiotherapy or radiofrequency ablation; (6) Patients who were HIV positive; and (7) Patients who were unable to tolerate adverse reactions.
