# Supplementary material for: Patient-derived gastric cancer organoids model heterogeneity and stroma-mediated chemoresistance in poorly cohesive carcinoma
Source: Front Mol Biosci. 2025 Jun 30;12:1631168. doi: 10.3389/fmolb.2025.1631168 (PMC12256257; doi:10.3389/fmolb.2025.1631168)
Supplement: Supplementary file 3 [file Table1.docx]

## Table S1

| **Antibodies and Reagents** | **Manufacturer, Country, Cat number, Lot number** | **Concentration** |
| --- | --- | --- |
| Dulbecco’s modified Eagle’s medium (DMEM) | Gibco, USA, C11995500BT, 8121032 | - |
| Phosphate buffered saline (PBS) | Gibco, USA, 70011-044, 8121322 | - |
| Fetal bovine serum (FBS) | Gibco, USA, 10099-141, 42F1376K | - |
| Matrigel | Corning, USA, 356231, 10124002 | - |
| Primary tissue storage solution | Bio Genous Technology, K6010005, 20240515 | - |
| Organoid recovery solution | Bio Genous Technology, E238006, 20240515 | - |
| Organoid dissociation solution | Bio Genous Technology, E238001, 20240515 | - |
| Red blood cell lysis solution | Bio Genous Technology, E238010, 20240515 | - |
| Tumor tissue digestion solution basal medium A | Bio Genous Technology, K601003-A100, 20240515 | - |
| Tumor tissue digestion solution basal medium B (20×) | Bio Genous Technology, K601008-B100, 20240515 | - |
| Gastric cancer Organoid Basal Medium A | Bio Genous Technology, K2179-GC- A500, 20240515 | - |
| Gastric cancer organoid kit | Bio Genous Technology, K2179-GC, 20240515 | - |
| LivingCell-Fluo™ Organoid Vitality Assay Kit | Bio Genous Technology, E238004 | - |
| 4',6-diamidino-2-phenylindole  (DAPI) | Beyotime Biotechnology, China, C1002,  091620210520 | - |
| Oxaliplatin | Jiangsu Hengrui Pharmaceuticals Co., Ltd. H20213312, 250127BV | 10mL/50mg |
| Irinotecan | Hainan Jinrui Pharmaceutical Co., Ltd. H20143126, 240804 | 5mL/100mg |
| 5-fluorouracil | Tianjin Jinyao Amino Acid Co., Ltd. H12020959, 2503201 | 10mL/250mg |
| Docetaxe | Jiangsu Hengrui Pharmaceuticals Co., Ltd. H20163032, 250126BV | 1mL/20mg |
| Anti-Pan-CK Rabbit polyclonal Antibody | ZSGB-BIO, China, ZM-0069 | IHC:1:200 |
| Anti-Ki67 Rabbit polyclonal Antibody | Cell Signaling Technology, USA, 34330 | IHC:1:200 IF:1:200 |
| Anti-CEA Rabbit polyclonal Antibody | Abcam plc, UK, ab300061 | IHC:1:200 IF:1:200 |
| Anti-CDX2 Mouse polyclonal Antibody | Abcam plc, UK, ab236536 | IHC:1:200 IF:1:200 |
| CoraLite488®-conjugated Goat Anti-Rabbit IgG(H+L) | Proteintech, China, SA00013-2, 205001014 | IF 1:300 |
| CoraLite594®-conjugated Goat Anti-Mouse IgG(H+L) | Proteintech, China, SA00013-3, 20000154 | IF 1:300 |
