# Supplementary figures and images for: Patient-derived gastric cancer organoids model heterogeneity and stroma-mediated chemoresistance in poorly cohesive carcinoma
Source: Front Mol Biosci. 2025 Jun 30;12:1631168. doi: 10.3389/fmolb.2025.1631168 (PMC12256257; doi:10.3389/fmolb.2025.1631168)

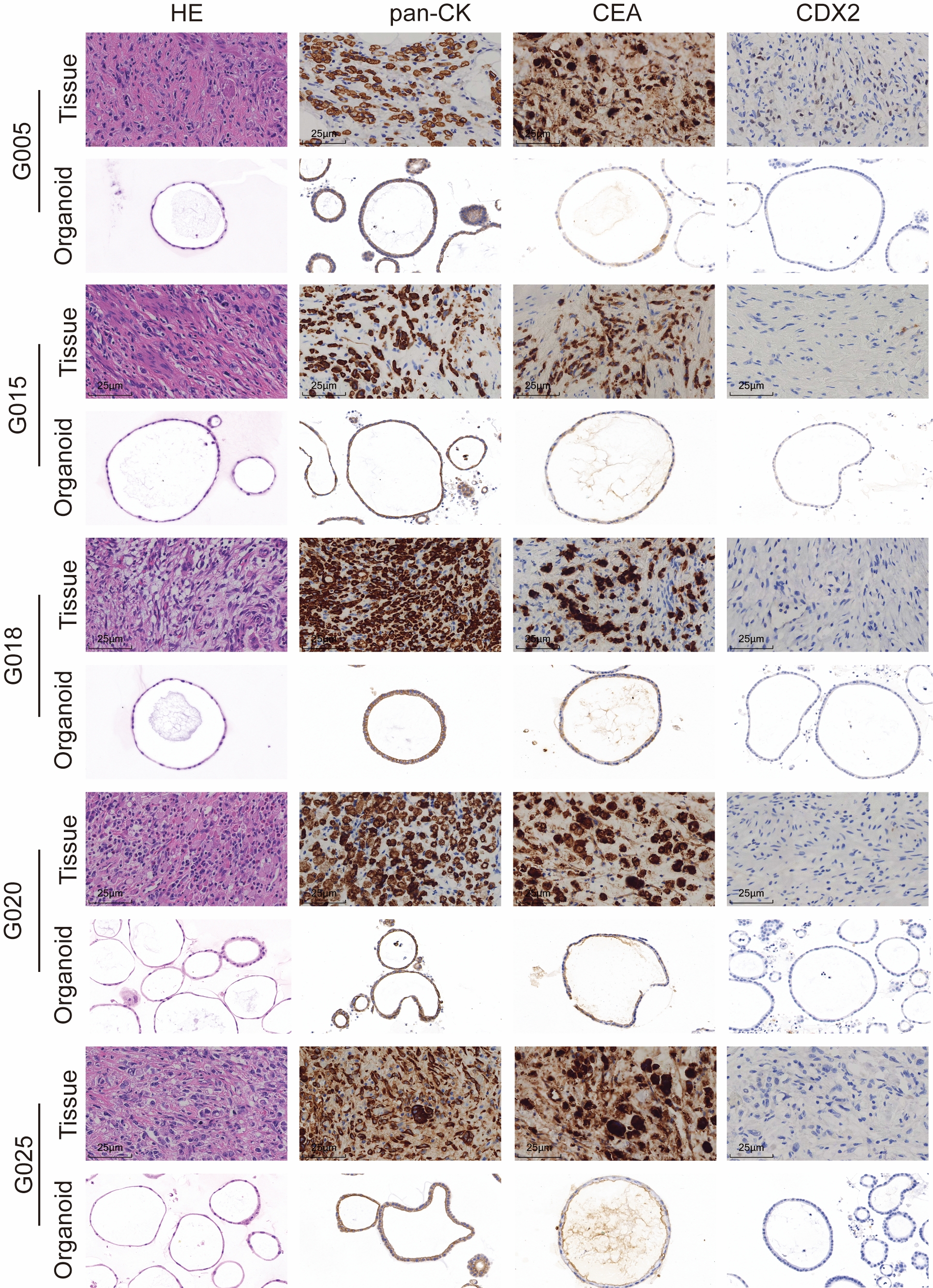

Supplement: Supplementary file 4 [file Image1.jpeg]

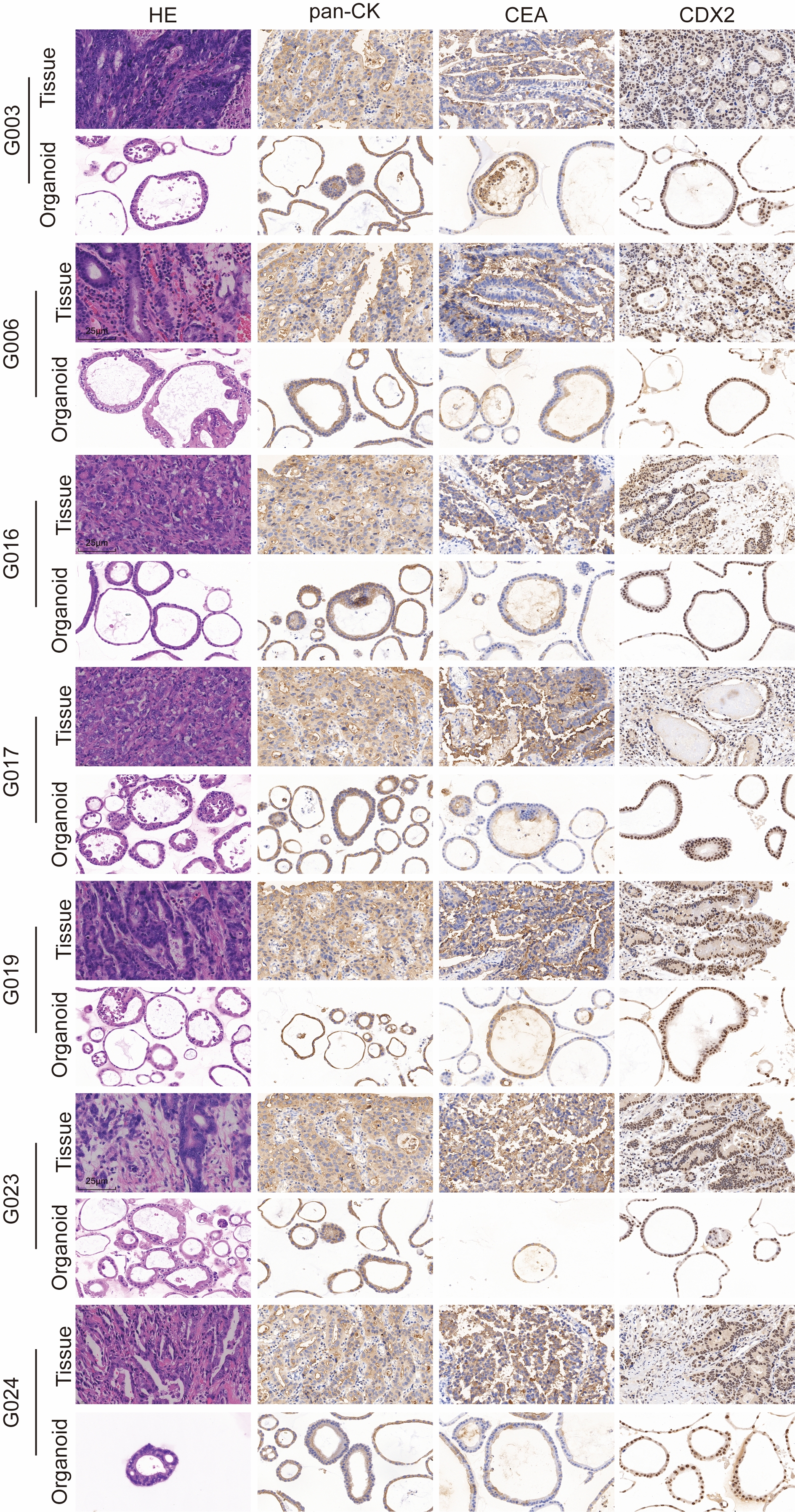

Supplement: Supplementary file 5 [file Image2.jpeg]
